# Supplementary material for: Lysozyme Fibrils Alter the Mechanism of Insulin Amyloid Aggregation
Source: Int J Mol Sci. 2021 Feb 10;22(4):1775. doi: 10.3390/ijms22041775 (PMC7916790; doi:10.3390/ijms22041775)
Supplement: Supplementary file 1 [file ijms-22-01775-s001.zip › ijms-1067159-supplementary/ijms-1067159-supplementary Figures.docx]

**Supplementary Materials**

Lysozyme Fibrils Alter the Mechanism of Insulin Amyloid
Aggregation

Mantas Ziaunys ^1^, Andrius Sakalauskas ^1^, Tomas Sneideris ^1,2^ and Vytautas Smirnovas ^1,^*

^1^ Life Sciences Center, Institute of Biotechnology, Vilnius University, LT-10257 Vilnius, Lithuania;
mantas.ziaunys@gmail.com (M.Z.); sakalauskas.and@gmail.com (A.S.); sneideris.t@gmail.com (T.S.)

^2^ Department of Chemistry, University of Cambridge, Cambridge CB2 1EW, UK

***** Correspondence: vytautas.smirnovas@bti.vu.lt

**Figure S1.** Aggregation of insulin in AC solution (20% acetic acid solution, containing 100 mM NaCl) with 0 µM (**A**), 2 µM (**B**), and 200 µM (**C**) lysozyme fibrils. Grey-color kinetic curves correspond to control samples, which do not contain insulin. Different color kinetic curves (shades of grey and green) represent three independent repeats.

**Figure S2.** Aggregation of insulin (200 µM) in AC solution with 0 µM (**A**), 2 µM (**B**), and 200 µM (**C**) lysozyme fibrils (control sample signal intensities are subtracted). The fluorescence intensity of ThT bound to insulin fibrils (**D**), normalized curve slope values (**E**), and aggregation half-time (t_50_) values (**F**). Fluorescence intensity differences, slope values, and t_50_ values were determined after subtracting the signal intensities of the control sample from the reaction sample. Different shades of green kinetic curves indicate three separate repeats.

**Figure S3.** Aggregation of insulin (200 µM) in AC solution with 0 µM (**A**), 2 µM (**B**), and 200 µM (**C**) lysozyme fibrils (control sample signal intensities are subtracted). The fluorescence intensity of ThT bound to insulin fibrils (**D**), normalized curve slope values (**E**), and aggregation half-time (t_50_) values (**F**). Fluorescence intensity differences, slope values, and t_50_ values were determined after subtracting the signal intensities of the control sample from the reaction sample. Different shades of green kinetic curves indicate three separate repeats.

**Figure S4.** Aggregation of insulin (200 µM) in AC solution with 2 µM of non-sonicated or sonicated lysozyme fibrils (**A**) (control sample signal intensities are subtracted). Normalized curve slope values (**B**), aggregation half-time (t_50_) values (**C**), and fluorescence intensity of ThT bound to insulin fibrils (**D**). Fluorescence intensity differences, slope values, and t_50_ values were determined after subtracting the signal intensities of the control sample from the reaction sample. Lysozyme fibrils were sonicated using the same method as insulin fibrils (as described in the Materials and Methods Section).

**Figure S5.** Aggregation of insulin (200 µM) in AC solution with 20 µM lysozyme fibrils. Grey-color kinetic curves correspond to control samples, which do not contain insulin. Different color kinetic curves (shades of grey and green) represent three independent repeats.

**Figure S6.** FTIR spectra of three batches of insulin aggregated in AC solution in the absence (**A**) or presence of 2 µM (**B**) and 200 µM lysozyme fibrils (**C**) and their second derivatives (**D**,**E,** and **F,** respectively). In cases when lysozyme fibrils were present in solution, their corresponding spectrum was subtracted from the mixture’s spectrum as described in the Materials and Methods Section.

**Figure S7.** Aggregation of insulin in AC solution in the absence (green) or presence (red) of 200 µM lysozyme monomers (**A**). Resulting fibril FTIR spectra (**B**) and second derivatives (**C**).

**Figure S8.** Seeded aggregation kinetics in the absence (**A**) and presence of 2 µM (**B**) and 200 µM (**C**) lysozyme fibrils. Grey-color kinetic curves correspond to control samples, which do not contain insulin. A representative curve is shown for every condition, additional repeats are available as Supplementary Material.

**Figure S9.** Insulin (200 µM) seeded aggregation kinetics in the absence (**A**) and presence of 2 µM (**B**) and 200 µM (**C**) lysozyme fibrils (control sample signal intensities are subtracted). Normalized curve slope values (**D**), aggregation half-time (t_50_) values (**E**), and fluorescence intensity of ThT bound to insulin fibrils (**F**). Fluorescence intensity differences, slope values, and t_50_ values were determined after subtracting the signal intensities of the control sample from the reaction sample. A representative curve is shown for every condition, additional repeats are available as Supplementary Material.

**Figure S10.** Insulin (200 µM) seeded aggregation kinetics in the absence (**A**) and presence of 2 µM (**B**) and 200 µM (**C**) lysozyme fibrils (control sample signal intensities are subtracted). Normalized curve slope values (**D**), aggregation half-time (t_50_) values (**E**), and fluorescence intensity of ThT bound to insulin fibrils (**F**). Fluorescence intensity differences, slope values, and t_50_ values were determined after subtracting the signal intensities of the control sample from the reaction sample. A representative curve is shown for every condition, additional repeats are available as Supplementary Material.

**Figure S11.** Bound-ThT fluorescence intensity of insulin fibril samples, prepared in the presence of 200 µM LF and 1%, 10^−4^% or 10^−8^% initial seed concentration, before and after sonication. Sample sonication was done by collecting the solutions from the 96-well plate and sonicating them using a MS-72 tip with 20% power for 20 s. Fluorescence intensity was measured at 25 °C. Intensity change percentage is indicated above each condition’s bar graphs.
